# Knowledge, Attitudes, and Practices Among Thoracic Healthcare Professionals Toward Postoperative Pulmonary Embolism

**DOI:** 10.3390/healthcare13151771

**Published:** 2025-07-22

**Authors:** Yuefeng Ma, Xin Xing, Shaomin Li, Jianzhong Li, Zhenchuan Ma, Liangzhang Sun, Danjie Zhang, Ranran Kong

**Affiliations:** 1 Department of Thoracic Surgery, The Second Affiliated Hospital of Xi’an Jiaotong University, Xi’an 710004, China; myf405@163.com (Y.M.); li51487@163.com (S.L.); jianzhong-0520@163.com (J.L.); gzuozhuanyong1@126.com (Z.M.); sunliangzhang@126.com (L.S.); renpengyu2001@163.com (D.Z.); 2 Cadre Health Care Special Clinic, The Second Affiliated Hospital of Xi’an Jiaotong University, Xi’an 710004, China; xx17187574@163.com

**Keywords:** knowledge, attitudes, practices, cross-sectional study, thoracic surgery, pulmonary embolism

## Abstract

**Background:** Postoperative pulmonary embolism (PPE) is a critical complication that can significantly affect patient outcomes. This study aimed to assess knowledge, attitudes, and practices (KAP) of thoracic healthcare professionals toward PPE. **Methods:** A cross-sectional study was conducted from September to December 2022. **Results:** A total of 222 thoracic healthcare professionals participated in the study; the majority were aged 30–40 years (40.54%) and had over 10 years of work experience (47.75%). Participants completed a self-designed questionnaire assessing demographic data and KAP scores: knowledge (0–11), attitudes (11–55), and practices (9–45). The main measures included the mean scores for knowledge, attitudes, and practices, along with correlation analyses and path analysis to assess relationships among the KAP components. Mean scores were 9.03 ± 1.13 for knowledge, 50.09 ± 4.23 for attitudes, and 35.78 ± 7.85 for practices. Participants showed strong awareness of PPE definitions and risk factors, but only 24.77% correctly identified its classic clinical triad. Attitudinally, while most expressed a willingness to engage in PPE training and risk assessment, 55.41% remained cautious about anticoagulation due to bleeding risks. In practice, although 72.52% consistently supported postoperative mobilization, only 30.63% frequently acquired updated PPE knowledge. Significant positive correlations were found between knowledge and attitudes (r = 0.218, *p* < 0.001) and between attitudes and practices (r = 0.234, *p* < 0.001). Path analysis showed that knowledge positively influenced attitudes (path coefficient 0.748, *p* = 0.002), and attitudes positively influenced practices (path coefficient 0.374, *p* = 0.003). **Conclusions:** Thoracic healthcare professionals exhibited adequate knowledge, positive attitudes, and proactive practices regarding PPE, indicating a strong foundation for enhancing postoperative care.

## 1. Introduction

Postoperative pulmonary embolism (PPE) ranks among the most severe postoperative thoracic surgery complications, reported in 1.5–11% of cases, but resulting in up to 30% of 30-day major surgery-specific case fatality rate [1,2,3]. Notable mortality is often attributable to insidious onset and high rate of misdiagnosis, despite rigorous adoption of thromboembolic prophylactic measures [1,4]. Prior to the occurrence of fatal outcomes, PPE often manifests with subtle symptoms like dyspnea and chest pain, frequently confounded with conditions such as congestive heart failure, sudden cardiac death, or pulmonary infections [4,5]. Furthermore, the underlying pathophysiology of thoracic surgical conditions and post-operative symptoms may obscure the clinical signs and manifestations of postoperative pulmonary embolism, creating additional challenges for healthcare professionals [1].

The prevention and management of PPE are of paramount significance in clinical practice, and healthcare professionals in thoracic surgery play a central role in this regard [6,7,8]. However, there are no universally adopted prophylaxis guidelines, and the certainty of the supporting evidence for many recommendations is low, due to the lack of direct evidence for thoracic surgery [9]. As a result, there might be notable gaps in understanding the current goals/methodology of PPE prevention, as well as the lack of communication between healthcare professionals and patients. Addressing those gaps with suitable educational interventions is essential to enhance patient care and mitigate the associated risks of PPE [10].

Knowledge, Attitudes, and Practices (KAP) study is a widely employed method in the medical domain for appraising knowledge levels, attitudes, and practical behaviors of healthcare professionals regarding specific subjects [11]. Within the medical realm, KAP studies have proven effective in evaluating the comprehension of healthcare professionals or patients towards various medical interventions and help to design new educational programs to address discovered barriers for better practice [12,13]. Studies conducted in China suggest that the limited attention to the prevention and management of PPE may be attributed to surgeons erroneously perceiving it as infrequent, in addition to the concerns regarding hemorrhagic risks and apprehensions about the increment in medical expenditures [14,15]. However, there exists a noticeable dearth of comprehensive research dedicated to assessing the knowledge and attitudes of thoracic surgery healthcare professionals concerning the prevention and management of PPE. Although some recent KAP studies have been conducted in thoracic surgical populations—such as those focused on enhanced recovery after surgery (ERAS) among thoracoscopy patients—there is still a lack of data specifically targeting thoracic healthcare professionals and their role in PPE prevention.

Previous KAP studies in healthcare settings have shown that insufficient knowledge often leads to inconsistent or incorrect practices, whereas positive attitudes are closely associated with better adherence to preventive measures and clinical guidelines [12,13]. This study employs the KAP framework to assess thoracic surgery healthcare professionals’ understanding of PPE definitions, as well as their knowledge, attitudes, and practices related to its treatment, with the aim of providing evidence-based insights and practical guidance for the effective management of postoperative pulmonary embolism.

## 2. Materials and Methods

### 2.1. Study Design

This cross-sectional study was conducted among thoracic surgery healthcare professionals from September to December 2022. Inclusion criteria were as follows: (1) age ≥ 18 years; (2) possession of relevant professional qualifications; and (3) routine involvement in PPE management. Participants included individuals from various professional roles (e.g., surgeons and nurses) and levels of expertise. Those who had previously participated in similar studies were excluded.

The study was ethically approved by the Medical Ethics Committee of the Second Affiliated Hospital of Xi’an Jiaotong University (Approval No. 2023043), and informed consent was obtained from the study participants.

### 2.2. Questionnaire

The questionnaire was developed based on the Chinese Expert Consensus on Perioperative Lung Protection in Thoracic Surgery (2019) [16]. The questionnaire draft was revised based on feedback from six senior thoracic surgery experts. A pilot test was conducted among 38 thoracic surgery healthcare professionals (including doctors and nurses) from the same institution to evaluate item clarity, content relevance, and overall feasibility. The pilot test yielded a Cronbach’s α coefficient of 0.823, indicating good internal consistency. Additionally, participants were invited to provide written and verbal feedback on the wording, length, and response options of the questionnaire. Based on this feedback, minor revisions were made to enhance clarity and consistency, particularly for items related to attitudes and practices. Although item-level statistical data from the pilot test were not retained, the pilot primarily served to improve quality and assess internal reliability.

The final questionnaire was in Chinese and comprised four parts: demographic information, knowledge, attitudes, and practices. The knowledge dimension comprised 11 questions, for which the right answers were awarded with 1 point, resulting in a possible score range of 0–11. The items related to attitudes and practices were presented in the form of a 5-point Likert scale. In the attitude dimension, positive statements (denoted as “P”) were assigned values from “a” to “e”, corresponding to scores from 5 to 1. Negative statements (denoted as “N”) were reverse-scored. Since the attitude dimension had 11 questions, the possible score range was from 11 to 55 points. The practice dimension was evaluated using nine questions scored on a 5-point Likert scale as well, with scores ranging from 9 to 45 points.

Data collection was conducted via the Sojump online platform (http://www.sojump.com, accessed on 17 November 2022). Using convenience sampling, questionnaire links were distributed to thoracic surgery departments in tertiary hospitals in Shaanxi Province to ensure diversity in clinical settings. Department heads were invited to forward the links to eligible thoracic surgeons and nurses. All data were collected anonymously. The online questionnaire system required all questions to be answered before submission to prevent missing values. As the questionnaire was distributed through an open link and forwarded by department heads without centralized tracking, the exact response rate could not be determined. IP address restrictions were implemented to prevent duplicate submissions, ensuring each unique IP address could submit the survey only once to maintain data consistency. A total of 224 questionnaires were collected, and after excluding 2 with anomalous responses in the occupation field, 222 were included in subsequent analyses.

### 2.3. Sample Size Calculation

Given the limited number of healthcare providers meeting the inclusion criteria, the sample size was determined based on the principle of 5–10 times the number of observed variables. The final questionnaire contained 31 items across the knowledge, attitude, and practice sections, indicating a required minimum sample size of approximately 155–310.

### 2.4. Statistical Analysis

Statistical analysis was performed using STATA 17.0 (StataCorp, College Station, TX, USA). Confirmatory factor analysis (CFA) and path analysis were conducted using AMOS 27.0 and SPSS 27.0 (IBM Corp., Armonk, NY, USA) to evaluate the validity of the questionnaire. Continuous variables were expressed as mean ± standard deviation (SD), with intergroup comparisons conducted using *t*-tests or analysis of variance (ANOVA). Categorical variables were presented as frequencies and percentages (n, %). Pearson correlation analysis was used to evaluate relationships between knowledge, attitude, and behavior scores. This study employed a structured approach to examine how knowledge and attitudes influence healthcare professionals’ behavioral practices, based on the following hypotheses: (1) Postoperative pulmonary embolism knowledge among thoracic surgery healthcare professionals directly affects their attitudes; (2) knowledge directly influences practices; and (3) attitudes directly affect practices. Path analysis, a specialized form of structural equation modeling (SEM), was utilized to analyze relationships among knowledge, attitudes, and practices. By simultaneously evaluating complex indirect effects among observable variables (knowledge, attitudes, and practices), path analysis addresses limitations of traditional regression methods. Unlike simple correlation or regression analyses, path analysis enables the examination of both direct and indirect pathways, providing a comprehensive understanding of the indirect effects of these factors. Model fit was evaluated using root mean square error of approximation (RMSEA < 0.08), standardized root mean square residual (SRMR < 0.08), Tucker–Lewis index (TLI > 0.80), and comparative fit index (CFI > 0.80). A two-sided *p*-value < 0.05 was considered statistically significant.

## 3. Results

**Figure 1:**
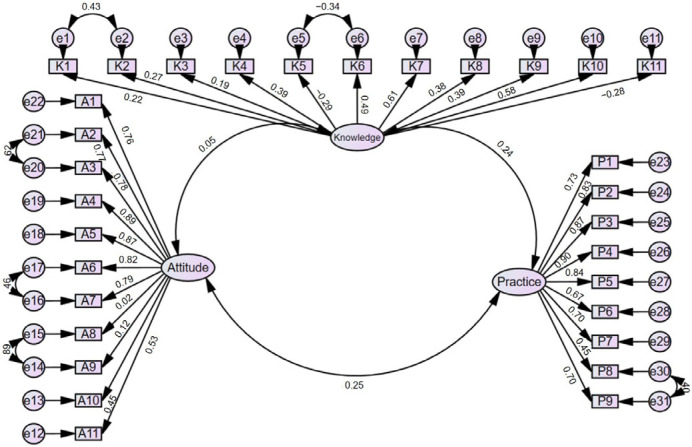
Confirmatory factor model.

This study initially collected 224 questionnaires. After excluding 2 with anomalous responses in the occupation field, 222 valid questionnaires were included in the analysis. The overall Cronbach’s α coefficient of the questionnaire was 0.840, indicating good internal consistency. The CFA results showed that RMSEA was 0.072, TLI was 0.856, IFI was 0.870, and CFI was 0.869, all of which meet accepted thresholds for a good model fit, thereby confirming the construct validity of the questionnaire (Figure 1 and Table S1).

### 3.1. Characteristics of the Participants

A total of 222 participants were included in this study; 90 (40.54%) of them were 30–40 years old, 106 (47.75%) had work experience of more than 10 years, 102 (45.95%) were physicians, and 120 (54.05%) were nurses. The mean knowledge, attitudes, and practices scores were 9.03 ± 1.13 (possible range: 0–11), 50.09 ± 4.23 (possible range: 11–55), and 35.78 ± 7.85 (possible range: 9–45), respectively. Notably, male participants showed significantly higher attitude and practice scores than females (*p* = 0.003 and *p* = 0.023, respectively). Participants with more than 10 years of experience also demonstrated higher practice scores (*p* = 0.006), while those with Master’s degrees exhibited more positive attitudes than other educational groups (*p* = 0.049). Surgeons had significantly higher scores in all three KAP dimensions compared to nurses, especially in attitudes (*p* = 0.004) and practices (*p* = 0.005) (Table 1).

### 3.2. Knowledge

The three knowledge items with the highest correctness rates were definition of PPE (K1) with a correctness rate of 98.65%, PPE risk factors (K4) with a correctness rate of 98.65%, and “Pulmonary embolism exhibits subtle clinical manifestations, a propensity for misdiagnosis, and alarmingly high mortality rates, warranting vigilant attention” (K3) with a correctness rate of 98.20%. The three items with the lowest correctness rates included clinical presentation of PPE (K5) with a correctness rate of 24.77%, surgical interventions (K11) with a correctness rate of 45.95%, and primary diagnostic modalities for PPE (K3) with a correctness rate of 75.23% (Table 2).

### 3.3. Attitudes

Most of the participants demonstrated positive attitudes toward all questions (Table 1). In particular, 99.55% of participants agreed or strongly agreed that it is crucial for medical professionals in thoracic surgery to undergo training regarding prevention and treatment of PPE (A1), 98.65% agreed or strongly agreed that it is imperative to assess the risk of pulmonary embolism in patients during the perioperative phase to ensure their well-being (A4), and 98.2% agreed or strongly agreed that proficiency in diverse treatment options for PPE is of paramount importance for the prognosis of thoracic surgery patients (A5). However, 55.41% claimed that they are cautious about anticoagulant interventions due to the potential risk of bleeding (A8), while 26.57% reported that after balancing the costs associated with various preventative and control measures for PPE after thoracic surgery, they are somewhat reluctant to implement these interventions (A9) (Table 3).

### 3.4. Practices

Most of the participants (88.74%) reported that they recommend or assist patients in post-surgery activities as part of PPE prevention and treatment (P8). More than half of the participants (66.21%) claimed that they employed multiple techniques to detect the occurrence of venous thrombosis in patients in clinical practice (P7). However, the participants rarely seek new knowledge (P1), with less than half (49.55%) claiming that they regularly acquire knowledge pertaining to the prevention and treatment of PPE. Furthermore, more than 40% of the participants reported that they never evaluated or documented PPE risk factors prior to the surgical procedure (P2) (Table 4).

### 3.5. Correlation Analysis

The Pearson’s correlation analysis indicated significant positive correlations between knowledge and attitudes (r = 0.218, *p* < 0.001), as well as attitudes and practices (r = 0.234, *p* < 0.001) (Table S2).

### 3.6. Path Analysis

**Figure 2:**
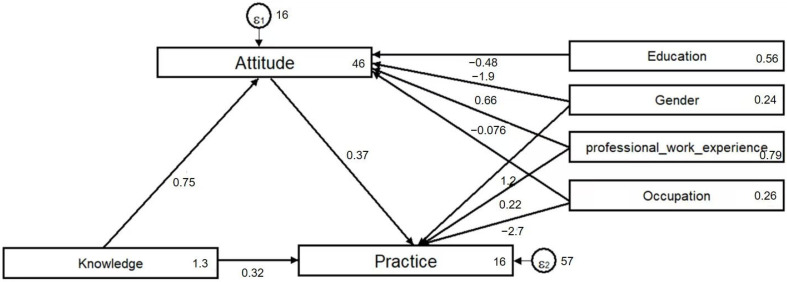
Path analysis model demonstrating the relationship between knowledge, attitudes, and practices.

A path analysis model was developed to investigate how thoracic surgery healthcare professionals’ knowledge and attitudes regarding PPE influence their behavioral practices and to examine whether attitudes mediate the relationship between knowledge and practices, as well as whether knowledge directly affects practices, consistent with the KAP theory (Figure 2). Hypothesis testing results are shown in Table S3. The model’s fit indices (RMSEA = 0.000, SRMR = 0.001, TLI = 1.365, CFI = 1.000) exceeded recommended thresholds, indicating an excellent fit between the model and the data (Table S4).

Path analysis results revealed that knowledge and work experience significantly and positively influenced attitudes and practices, with path coefficients of 0.748 (*p* = 0.002) and 0.660 (*p* = 0.032), respectively. The model further demonstrated that attitudes significantly and positively affected practices, with a path coefficient of 0.374 (*p* = 0.003). As the model was constructed using observed variables (total scores for knowledge, attitudes, and practices), no separate measurement model for latent constructs was evaluated. Prior to path analysis, variable distributions were examined, and all KAP scores exhibited approximate normal distribution, with skewness and kurtosis values within acceptable ranges. The sample size of 222 met the commonly recommended threshold for path analysis (e.g., ≥200), ensuring model stability. Although confidence intervals for fit indices were not calculated, the model demonstrated excellent fit (RMSEA = 0.000, SRMR = 0.001, TLI = 1.365, CFI = 1.000), supporting the hypothesized relationships. These findings suggest that enhancing knowledge among thoracic surgery healthcare professionals may foster more positive attitudes, thereby improving clinical practices related to PPE prevention. The strong path coefficient of knowledge on attitudes (0.748) indicates that knowledge is a foundational factor in shaping beliefs and perceptions. The observed indirect pathway, whereby knowledge influences practices through attitudes, underscores the importance of comprehensive educational interventions targeting both knowledge and attitudes, which may drive behavioral changes in clinical settings.

### 3.7. Stratification Analysis

Knowledge scores did not differ between participants with different epidemiological characteristics (all *p* > 0.05), but attitude scores were significantly higher attitudes in male participants (*p* = 0.003), those with work experience spanning 6–9 years (*p* = 0.015), in possession of a Master’s degree (*p* = 0.049), and surgeons (*p* = 0.015). Male participants (*p* = 0.023) and those with work experience of more than 10 years (*p* = 0.006) exhibited higher practice scores.

Since there was only one nursing assistant involved in the study, this study focused on differences only between surgeons and nurses in the stratified analysis, and the nursing assistant was excluded (Tables S4–S6). Key findings from the Supplementary Tables showed that surgeons had significantly higher knowledge regarding the clinical triad of PPE (33.3% vs. 17.5%, *p* = 0.006), while nurses showed better awareness of diagnostic tools such as Doppler ultrasound (82.5% vs. 66.7%, *p* = 0.006) (Table S5). Attitudinally, surgeons were more positive regarding anticoagulant use (65.69% vs. 46.67%, *p* = 0.004) and less concerned about cost (72.55% vs. 59.17%, *p* = 0.037) (Table S6). For practice, surgeons reported significantly more frequent engagement in active knowledge acquisition and risk factor evaluation compared to nurses (P1: 58.82% vs. 41.67%, *p* = 0.011; P2: 67.65% vs. 51.67%, *p* = 0.016) (Table S7). These subgroup findings support the observed differences in KAP scores and indicate areas for targeted improvement.

In terms of knowledge, the correctness rate for identifying the PPE triad and bedside color Doppler ultrasound was lower than other questions (K5), with only 33.3% of surgeons and 17.5% of nurses correctly identifying the PPE triad, with the correct rate of surgeons higher than that of nurses (*p* = 0.006). In contrast, color Doppler ultrasound and transesophageal echocardiography, as the first-choice screening methods for PPE, had a high correct rate among nurses (82.5% vs. 66.7%, *p* = 0.006).

Regarding attitude, the positive rate of surgeons’ attitudes towards the prophylactic use of anticoagulation in patients after thoracic surgery was higher than that of nurses (65.69% vs. 46.67%, *p* = 0.004). Similarly, surgeons had a more positive attitude towards the possible cost of prevention and treatment of PPE after thoracic surgery compared to nurses (72.55% vs. 59.17%, *p* = 0.035).

In terms of practice, surgeons had significantly higher positive rates than nurses in knowledge acquisition, symptoms observation, and anesthetic-related risk assessment related to PPE prevention and management (question P1, 58.82% vs. 41.67%, *p* = 0.011; question P2, 67.65% vs. 51.67%, *p* = 0.016; question P4, 69.61% vs. 56.67%, *p* = 0.047, respectively).

## 4. Discussion

In this study, thoracic healthcare professionals demonstrated sufficient knowledge, positive attitudes, and proactive practices toward pulmonary embolism. Areas that need improvement were identified, including recognition of PPE clinical presentation and diagnostic modalities, as well as addressing concerns about the costs and potential risks of prophylaxis measures. The implementation of training and educational programs can effectively improve healthcare professionals’ understanding of PPE, cultivate a positive outlook, and enhance procedural efficiency.

The study evaluated knowledge of thoracic healthcare professionals regarding PPE and found comparatively high knowledge scores among participants, with a mean score of 9.03 out of 11. Although the study design does not allow direct comparison, it could be noted that previous studies undertaken among patients and nurses demonstrated low knowledge scores with the urgent need for improvement [17,18,19]. In the present study, most participants correctly answered questions related to the definition of PPE, risk factors, and diagnostic modalities. However, the recognition of clinical manifestations of PPE (K5) yielded a lower correct response rate, consistent with a previous study conducted in the Netherlands. In that study, patients experienced an average total delay of 8.6 days, highlighting the substantial diagnostic delay, particularly attributed to patient delay and primary care delay [20]. This underscores the potential for mitigating this delay by enhancing awareness among healthcare professionals through targeted educational interventions.

The attitudes of thoracic healthcare professionals towards PPE were generally positive, in line with other studies conducted among healthcare professionals regarding thromboembolism prophylaxis [21,22,23]. Compared to nurses, surgeons demonstrated higher attitude scores; moreover, seemingly significant gender differences in attitude are most likely also attributable to the professional differences, as most nurses in this study were female and most surgeons were male. This overlap suggests that gender and occupational role may have a compounded effect on KAP, potentially influenced by differences in training opportunities, clinical responsibilities, and confidence in handling complex clinical scenarios such as PPE. To address these disparities, targeted interventions such as tailored training modules for nurses, particularly early-career female staff focusing on risk assessment, guideline familiarity, and anticoagulant management, could enhance confidence and alignment with best practices. Encouraging interdisciplinary discussions may also bridge the gap in perspectives between professional groups. More comprehensive education directed at less experienced nurses might help to bridge the gap and empower attitude changes. Still, most participants strongly agreed with statements emphasizing the importance of training, discussion with colleagues, and continuous learning to improve the standard of care. These findings are consistent with international research. For example, a multi-center study in Saudi Arabia reported that while healthcare professionals generally showed high awareness of VTE prevention guidelines, practical adherence remained inconsistent, particularly among nurses [21]. Similarly, a systematic review of nurses’ KAP toward VTE in various countries identified significant gaps in practice despite adequate knowledge and attitudes [22]. These parallels suggest that the gap between knowledge and actual practice is a common global challenge, reinforcing the need for targeted interventions and system-level support. However, some healthcare professionals expressed concerns about the costs associated with preventive and control measures and the potential risks of anticoagulant interventions, particularly heparin (A8, 9). These concerns may be addressed through enhanced education about the favorable risk–benefit profile of thromboprophylaxis, as supported by guidelines, which emphasize that, when appropriately applied, anticoagulant strategies significantly reduce morbidity and mortality with manageable bleeding risk. Adherence to well-established clinical guidelines, such as those from the American College of Chest Physicians and the Joint Thoracic Surgery Association, has been shown to improve the safety and efficacy of thromboprophylaxis, thereby addressing provider hesitations [24]. Moreover, a study has demonstrated the cost-effectiveness of thromboprophylaxis strategies in reducing overall healthcare burden by preventing severe thromboembolic complications and associated treatments [25]. A study investigating the management of venous thromboembolism (VTE) has elucidated the role of physician knowledge, attitudes, and beliefs in influencing clinical practices, thereby contributing to the observed variance from international guidelines. This study highlights the importance of healthcare facilities assessing their alignment with global VTE treatment guidelines and formulating approaches to enhance the administration of antithrombotic therapies [25]. Findings of the present study suggest the need to address these concerns and provide a balanced perspective on the benefits and risks of interventions.

The results of the practice dimension indicated that there is room for improvement in several areas, drawing attention to the substantial proportion of participants who reported inconsistent practices in actively acquiring knowledge (P1), evaluating risk factors in patients (P2), and monitoring symptoms of PPE (P3). Moreover, years of professional work experience had a notable impact on practices, with healthcare professionals having more than 10 years of experience showing more consistent practices. This underscores the importance of experience and seniority in shaping healthcare professionals’ practices, discussed in previous studies [26,27]. Furthermore, education levels and occupation did not exhibit significant effects on KAP scores, suggesting that formal education and occupation may not be strong determinants of KAP related to PPE, while regular and consistent training sessions and supervision of more experienced colleagues might help in improving KAP. The subgroup analysis revealed significant disparities between surgeons and nurses, particularly in their recognition of PPE symptoms, attitudes toward anticoagulation, and frequency of preventive practices. Surgeons demonstrated a higher correct response rate regarding the clinical triad of PPE, while nurses were more familiar with certain diagnostic tools, such as Doppler ultrasound. These differences likely reflect variation in clinical responsibilities, training focus, and decision-making autonomy. Additionally, surgeons showed greater acceptance of the cost and risk trade-offs associated with anticoagulant use. These findings suggest that intervention strategies should be profession-specific, enhancing nurses’ symptom recognition and decision confidence, while reinforcing guideline-based standardization across all groups. The discrepancy between significant occupation-based differences and the lack of clear educational effects may be explained by role-specific clinical duties and experiential learning. Surgeons generally receive more hands-on exposure to perioperative thrombo-prophylaxis decision-making and are directly accountable for its outcomes, whereas nurses often focus on implementation and monitoring. Such workplace factors, rather than formal degree level, may therefore drive the observed gaps in knowledge application and practice. The path analysis results also support the importance of fostering a positive attitude among thoracic healthcare professionals regarding PPE, as it can lead to improved knowledge and, more importantly, consistent practices in the prevention and treatment of PPE.

Nevertheless, the previous study had limitations. The primary concern pertains to the use of self-reported data, which introduces the potential for recall bias and subjectivity, potentially affecting the accuracy of responses. In addition, the cross-sectional design limits the ability to infer causal relationships between knowledge, attitudes, and practices. The sample was drawn from a single geographical area using convenience-based digital distribution, which may affect the generalizability of the findings. Furthermore, while subgroup analyses were conducted, some professional subcategories (e.g., senior staff and certain hospital levels) had relatively small sample sizes, which may have influenced statistical power and representation. The data collection occurred over a limited three-month period, which may have introduced selection bias related to seasonal variations in clinical workload, staffing schedules, or departmental operations. This may have influenced both participation rates and the representativeness of responses. Moreover, the number of participants from primary (n = 6) and secondary (n = 15) hospitals was very limited, reducing the reliability of comparative analyses across hospital tiers. This imbalance may introduce bias and restrict meaningful interpretation of hospital-level differences in KAP scores. Additionally, the relatively small sample size could restrict the broader generalizability of the study’s conclusions. Nevertheless, given the diversity in participant characteristics, future studies should consider formal sample size estimation based on hypothesized effect sizes to enhance statistical robustness and subgroup comparisons. Additionally, the structural validity and quantitative content validity of the questionnaire were not assessed, limiting conclusions about its comprehensive or representative coverage of the research domain. Future studies should include thorough validation to address this limitation. Additionally, the limited variation in KAP scores across different educational levels may suggest insufficient discriminatory power of the questionnaire. As many knowledge items focused on basic clinical facts, they may lack the complexity needed to detect differences in critical thinking or higher-order understanding. Future assessments should incorporate more challenging or case-based items to enhance the questionnaire’s ability to differentiate across diverse educational and professional backgrounds.

## 5. Conclusions

In summary, thoracic healthcare professionals demonstrated sufficient knowledge, positive attitudes, and proactive practices toward pulmonary embolism. The findings indicate that targeted training programs could be explored as a potential strategy to address observed gaps in knowledge and attitudes concerning PPE, particularly among specific professional groups. New policies should be implemented to support such educational initiatives and consider the provision of resources to overcome the concerns related to the costs and risks of preventive measures.

## Figures and Tables

**Table 1:** Demographic characteristics and KAP scores.

Variables	N (%)	Knowledge Score		Attitude Score		Practices Score	
		Mean ± SD	*p*	Mean ± SD	*p*	Mean ± SD	*p*
**Total**	222	9.03 ± 1.13		50.09 ± 4.23		35.78 ± 7.85	
**Gender**			0.281		0.003 *		0.023 *
Male	95 (42.79)	9.14 ± 1.02		51.06 ± 3.86		36.96 ± 8.12	
Female	127 (57.21)	8.94 ± 1.20		49.37 ± 4.36		34.90 ± 7.55	
**Age**			0.26		0.061		0.138
<30 years	85 (38.29)	9.02 ± 0.91		49.28 ± 4.63		34.53 ± 8.07	
30–40 years	90 (40.54)	8.91 ± 1.40		50.29 ± 4.00		36.11 ± 7.92	
>40 years	47 (21.17)	9.26 ± 0.87		51.19 ± 3.66		37.40 ± 7.06	
**Years of professional work experience**			0.138		0.015 *		0.006 *
≤5 years	74 (33.33)	8.99 ± 0.93		49.03 ± 4.88		35.24 ± 8.40	
6–9 years	42 (18.92)	9.17 ± 1.06		50.69 ± 3.83		34.95 ± 7.24	
≥10 years	106 (47.75)	9.00 ± 1.28		50.60 ± 3.76		36.48 ± 7.69	
**Education**			0.674		0.049 *		0.185
Junior college and bachelor’s degree	137 (61.71)	8.99 ± 1.20		49.66 ± 4.31		35.14 ± 7.70	
Master’s degree	50 (22.52)	9.16 ± 1.00		51.28 ± 4.06		36.86 ± 8.69	
Doctorate	35 (15.77)	8.97 ± 1.01		50.09 ± 3.94		36.74 ± 7.07	
**Occupation**			0.207		0.004		0.005
Surgeons	102 (45.95)	9.14 ± 0.98		50.95 ± 3.94		37.19 ± 7.96	
Nurses	120 (54.05)	8.92 ± 1.23		49.36 ± 4.34		34.57 ± 7.57	
**Professional title**			0.333		0.111		0.197
Primary	94 (42.34)	9.01 ± 1.02		49.77 ± 4.42		34.98 ± 8.24	
Middle	86 (38.74)	8.92 ± 1.34		49.76 ± 4.40		35.78 ± 7.94	
Vice-senior	25 (11.26)	9.28 ± 0.54		52.00 ± 2.74		36.16 ± 6.82	
Senior	17 (7.66)	9.29 ± 1.16		50.82 ± 3.41		39.65 ± 5.62	
**Hospital level**			0.137		0.088		0.768
Primary	6 (2.70)	8.33 ± 0.82		46.50 ± 3.51		34.17 ± 8.66	
Secondary	15 (6.76)	9.20 ± 0.56		50.40 ± 4.67		35.13 ± 8.02	
Tertiary	201 (90.54)	9.03 ± 1.16		50.18 ± 4.19		35.88 ± 7.85	

Note: * *p* < 0.05 was considered statistically significant.

**Table 2:** Knowledge.

	Correctness N (%)
K1. Pulmonary embolism encompasses a spectrum of conditions resulting from the occlusion of the pulmonary arterial system by emboli of diverse origins. It constitutes a significant postoperative complication in the realm of thoracic surgery.	219 (98.65)
K2. Chest trauma is a known factor contributing substantially to the heightened incidence of pulmonary embolism. This association may be attributed to the impeding of pulmonary circulation and concurrent lung tissue injury.	216 (97.3)
K3. Pulmonary embolism exhibits subtle clinical manifestations, a propensity for misdiagnosis, and alarmingly high mortality rates, warranting vigilant attention.	218 (98.2)
K4. Predisposing risk factors for post-thoracic surgery pulmonary embolism typically comprise advanced age, a history of smoking, obesity, trauma, and thoracic malignancies.	219 (98.65)
K5. The characteristic clinical presentations of pulmonary embolism, which typically manifest concomitantly, constitute the “pulmonary embolism triad,” encompassing dyspnea, chest pain, hemoptysis, and/or circulatory failure.	55 (24.77)
K6. Bedside color Doppler echocardiography, lower extremity vascular ultrasonography, and transesophageal echocardiography are often employed as the primary diagnostic modalities for pulmonary embolism.	167 (75.23)
K7. Spiral CT pulmonary angiography stands as a reliable method for guiding thrombolytic therapy and evaluating its therapeutic efficacy.	200 (90.09)
K8. Postoperative preventative measures encompass early mobilization, intermittent sequential compression to enhance lower limb blood circulation, and pharmaceutical prophylaxis, such as unfractionated heparin, low-molecular-weight heparin, and warfarin.	216 (97.3)
K9. Given the absence of anticoagulation contraindications, heparin anticoagulation represents the primary treatment modality for post-thoracic surgery pulmonary embolism.	204 (91.89)
K10. Interventional strategies for managing pulmonary embolism following thoracic surgery frequently entail a combination of mechanical thrombectomy, thrombectomy, and localized thrombolysis.	188 (84.68)
K11. Surgical intervention typically involves pulmonary artery thrombectomy, with early postoperative recovery demonstrating limited associations with overall prognosis.	102 (45.95)

**Table 3:** Attitudes.

	Strongly Agree N (%)	Agree N (%)	Neutral N (%)	Disagree N (%)	Strongly Disagree N (%)
A1. It is crucial for medical professionals in thoracic surgery to undergo training pertaining to the prevention and treatment of pulmonary embolism, as it directly impacts the execution of clinical responsibilities and patient prognosis. (P)	206 (92.79)	15 (6.76)	1 (0.45)	/	/
A2. You are open to discussing the challenges encountered during the clinical practice of pulmonary embolism following thoracic surgery with fellow healthcare practitioners and actively seeking viable solutions. (P)	195 (87.84)	26 (11.71)	1 (0.45)	/	/
A3. You are committed to acquiring expert consensus on pulmonary embolism following thoracic surgery, updating relevant knowledge, and enhancing the standard of care in preventing and treating pulmonary embolism. (P)	195 (87.84)	26 (11.71)	1 (0.45)	/	/
A4. Recognizing the latent and deleterious nature of pulmonary embolism symptoms post-thoracic surgery, it is imperative to assess the risk of pulmonary embolism in patients during the perioperative phase to ensure their well-being. (P)	197 (88.74)	22 (9.91)	3 (1.35)	/	/
A5. Proficiency in diverse treatment options for pulmonary embolism is of paramount importance for the prognosis of thoracic surgery patients, emphasizing the significance of medical staff in this specialty. (P)	192 (86.49)	26 (11.71)	4 (1.8)	/	/
A6. You acknowledge the significance of preoperative assessment for identifying risk factors related to pulmonary embolism in patients. (P)	192 (86.49)	28 (12.61)	2 (0.9)	/	/
A7. Early intervention in cases of pulmonary embolism is a pivotal factor in safeguarding the prognosis and quality of life for patients. (P)	189 (85.14)	32 (14.41)	1 (0.45)	/	/
A8. You are cautious about anticoagulant interventions due to the potential risk of bleeding when using heparin and other medications to prevent and treat pulmonary embolism post-thoracic surgery. (N)	60 (27.03)	63 (28.38)	38 (17.12)	10 (4.5)	51 (22.97)
A9. Balancing the costs associated with various preventative and control measures for pulmonary embolism after thoracic surgery, you are somewhat reluctant to implement these interventions. (N)	48 (21.62)	11 (4.95)	18 (8.11)	70 (31.53)	75 (33.78)
A10. You recognize the necessity of perfecting preoperative color Doppler ultrasound examinations for both lower extremities. (P)	150 (67.57)	56 (25.23)	9 (4.05)	5 (2.25)	2 (0.9)
A11. You have placed significant emphasis on monitoring D-dimer levels in the coagulation profile. (P)	156 (70.27)	50 (22.52)	12 (5.41)	3 (1.35)	1 (0.45)

**Table 4:** Practices.

	Very Consistent N (%)	Somewhat Consistent N (%)	Neutral N (%)	Somewhat Inconsistent N (%)	Very Inconsistent N (%)
P1. How frequently do you actively acquire knowledge pertaining to the prevention and treatment of pulmonary embolism following thoracic surgery through various means, such as engaging in training, reviewing medical literature or expert consensus, and engaging in discussions with fellow medical professionals? (P)	68 (30.63)	42 (18.92)	76 (34.23)	34 (15.32)	2 (0.9)
P2. How often do you evaluate or document risk factors or indications of pulmonary embolism in patients following thoracic surgery prior to the surgical procedure? (P)	81 (36.49)	50 (22.52)	50 (22.52)	37 (16.67)	4 (1.8)
P3. Throughout the course of thoracic surgery, do you conscientiously monitor variables including surgical duration, hematoma compression, and other intraoperative factors, and integrate them into your subsequent practices for the prevention and treatment of pulmonary embolism in patients? (P)	85 (38.29)	55 (24.77)	47 (21.17)	26 (11.71)	9 (4.05)
P4. While performing thoracic surgery, it is essential to maintain a heightened awareness of high-risk factors associated with pulmonary embolism linked to anesthesia, such as limb hypoperfusion and reduced venous blood flow, and incorporate these considerations into your preventative and treatment measures for embolization. (P)	85 (38.29)	54 (24.32)	47 (21.17)	26 (11.71)	10 (4.5)
P5. How closely do you monitor the frequency of symptoms related to pulmonary embolism, such as the pulmonary embolism triad, in post-surgery patients? (P)	100 (45.05)	61 (27.48)	41 (18.47)	17 (7.66)	3 (1.35)
P6. What is the frequency of your clinical engagement in preventative and treatment practices for pulmonary embolism using medications like heparin and warfarin for patients? (P)	115 (51.8)	50 (22.52)	35 (15.77)	16 (7.21)	6 (2.7)
P7. Have you employed techniques such as color Doppler echocardiography and lower limb vascular ultrasonography, as well as other imaging methods, to detect the occurrence of venous thrombosis in patients? (P)	92 (41.44)	55 (24.77)	41 (18.47)	17 (7.66)	17 (7.66)
P8. How frequently do you recommend or assist patients in post-surgery activities such as mobilization, local limb massage, regular repositioning, and elevation of the lower limbs as part of pulmonary embolism prevention and treatment? (P)	161 (72.52)	36 (16.22)	15 (6.76)	7 (3.15)	3 (1.35)
P9. Summarize your experience in the prevention and treatment of pulmonary embolism, encompassing preoperative assessment, intraoperative vigilance, and postoperative care. Apply this experience to enhance the frequency of pulmonary embolism prevention and treatment following thoracic surgery in the future. (P)	115 (51.8)	53 (23.87)	35 (15.77)	15 (6.76)	4 (1.8)

## Data Availability

The data presented in this study are available on request from the corresponding author due to ethical restrictions and patient confidentiality.

## Supplementary Materials

The following supporting information can be downloaded at: https://www.mdpi.com/article/10.3390/healthcare13151771/s1, Table S1: Confirmatory factor model results; Table S2: Pearson’s analysis; Table S3: Test results of the hypothesis; Table S4: Model fitness indices for the KAP structural equation model; Table S5: Correct rate of surgeons’ and nurses’ knowledge dimension of prevention and cure of PPE after thoracic surgery; Table S6: Positive rate of the attitude of surgeons and nurses to prevention and cure of PPE after thoracic surgery; Table S7: Positive rate of practices of surgeons and nurses in the prevention and cure of PPE after thoracic surgery.
