# Supplementary material for: Knowledge, Attitudes, and Practices Among Thoracic Healthcare Professionals Toward Postoperative Pulmonary Embolism
Source: Healthcare (Basel). 2025 Jul 22;13(15):1771. doi: 10.3390/healthcare13151771 (PMC12346016; doi:10.3390/healthcare13151771)
Supplement: Supplementary file 1 [file healthcare-13-01771-s001.zip › healthcare-3634322-supplementary.pdf]

Table S1. Confirmatory factor model results

Indicators	Reference	Actual
CMIN/DF	1-3: Excellent, 3-5: Good	2.168
RMSEA	<0.08: Good	0.072
IFI	>0.8: Good	0.870
TLI	>0.8: Good	0.856
CFI	>0.8: Good	0.869

CMIN/DF: Chi-square/Degrees of Freedom, RMSEA: Root Mean Square Error of Approximation; IFI: Incremental Fit Index; TLI: Tucker-Lewis Index; CFI: Comparative Fit Index

Table S2. Pearson's analysis

	Knowledge	Attitudes	Practices
Knowledge	1		
Attitudes	0.218 (P=0.001)	1	
Practices	0.107 (P=0.112)	0.234 (P<0.001)	1

Table S3. Test results of the hypothesis

		Estimate	P>|z|
Attitude <-			
	Knowledge	0.748	0.002
	Gender	-1.863	0.161
	professional_work_experience	0.660	0.032
	Education	-0.481	0.391
	Occupation	-0.076	0.959
Practice <-			
	Attitude	0.374	0.003
	Knowledge	0.317	0.497
	Gender	1.163	0.645
	professional_work_experience	0.216	0.712
	Occupation	-2.664	0.283

Table S4. Model fitness indices for the KAP structural equation model

Indicators	Reference	Results
RMSEA	<0.08 Good	0.000
SRMR	<0.08 Good	0.001
TLI	>0.8 Good	1.365
CFI	>0.8 Good	1.000

RMSEA: root mean square error of approximation; TLI: Tucker-Lewis index; CFI: comparative fix index.

Table S5. Correct rate of surgeons' and nurses' knowledge dimension of prevention and cure of PPE after thoracic surgery

	Surgeons (n=102)	Nurses (n=119)	P
K1. Pulmonary embolism encompasses a spectrum of conditions resulting from the occlusion of the pulmonary arterial system by emboli of diverse origins. It constitutes a significant postoperative complication in the realm of thoracic surgery.	102 (100.00)	117(97.5)	0.252
K2. Chest trauma is a known factor contributing substantially to the heightened incidence of pulmonary embolism. This association may be attributed to the impeding of pulmonary circulation and concurrent lung tissue injury.	99 (97.06)	117(97.5)	1.000
K3. Pulmonary embolism exhibits subtle clinical manifestations, a propensity for misdiagnosis, and alarmingly high mortality rates, warranting vigilant attention.	101 (99.02)	117(97.5)	0.627
K4. Predisposing risk factors for post-thoracic surgery pulmonary embolism typically comprise advanced age, a history of smoking, obesity, trauma, and thoracic malignancies.	102 (100.00)	117(97.5)	0.252

K5. The characteristic clinical presentations of pulmonary embolism, which typically manifest concomitantly, constitute the "pulmonary embolism triad," encompassing dyspnea, chest pain, hemoptysis, and/or circulatory failure.	34 (33.33)	21(17.5)	0.006
K6. Bedside color Doppler echocardiography, lower extremity vascular ultrasonography, and transesophageal echocardiography are often employed as the primary diagnostic modalities for pulmonary embolism.	68 (66.67)	99(82.5)	0.006
K7. Spiral CT pulmonary angiography stands as a reliable method for guiding thrombolytic therapy and evaluating its therapeutic efficacy.	94 (92.16)	106(88.33)	0.342
K8. Postoperative preventative measures encompass early mobilization, intermittent sequential compression to enhance lower limb blood circulation, and pharmaceutical prophylaxis, such as unfractionated heparin, low molecular weight heparin, and warfarin.	101 (99.02)	115(95.83)	0.222
K9. Given the absence of anticoagulation contraindications, heparin anticoagulation represents the primary treatment modality for post-thoracic surgery pulmonary embolism.	94 (92.16)	110(91.67)	0.894

K10. Interventional strategies for managing pulmonary embolism following thoracic surgery frequently entail a combination of mechanical thrombectomy, thrombectomy, and localized thrombolysis.	90 (88.24)	98(81.67)	0.176
K11. Surgical intervention typically involves pulmonary artery thrombectomy, with early postoperative recovery demonstrating limited associations with overall prognosis.	48 (47.06)	54(45)	0.759

Table S6. Positive rate of the attitude of surgeons and nurses to prevention and cure of PPE after thoracic surgery

	**Surgeons (n=102)**	**Nurses (n=119)**	**P**
A1. It is crucial for medical professionals in thoracic surgery to undergo training pertaining to the prevention and treatment of pulmonary embolism, as it directly impacts the execution of clinical responsibilities and patient prognosis. (P)	102 (100.00)	119 (99.17)	1.000
A2. You are open to discussing the challenges encountered during the clinical practice of pulmonary embolism following thoracic surgery with fellow healthcare practitioners and actively seeking viable solutions. (P)	102 (100.00)	119 (99.17)	1.000
A3. You are committed to acquiring expert consensus on pulmonary embolism following thoracic surgery, updating relevant knowledge, and enhancing the standard of care in preventing and treating pulmonary embolism. (P)	102 (100.00)	119 (99.17)	1.000
A4. Recognizing the latent and deleterious nature of pulmonary embolism symptoms post thoracic surgery, it is imperative to assess the risk of pulmonary embolism in patients during the perioperative phase to ensure their well-being. (P)	101 (99.02)	118(98.33)	1.000

A5. Proficiency in diverse treatment options for pulmonary embolism is of paramount importance for the prognosis of thoracic surgery patients, emphasizing the significance of medical staff in this specialty. (P)	100 (98.04)	118(98.33)	1.000
A6. You acknowledge the significance of preoperative assessment for identifying risk factors related to pulmonary embolism in patients. (P)	101 (99.02)	119 (99.17)	1.000
A7. Early intervention in cases of pulmonary embolism is a pivotal factor in safeguarding the prognosis and quality of life for patients. (P)	102 (100.00)	119 (99.17)	1.000
A8. You are cautious about anticoagulant interventions due to the potential risk of bleeding when using heparin and other medications to prevent and treat pulmonary embolism post thoracic surgery.(N)	67 (65.69)	56(46.67)	0.004
A9. Balancing the costs associated with various preventative and control measures for pulmonary embolism after thoracic surgery, you are somewhat reluctant to implement these interventions.(N)	74 (72.55)	71(59.17)	0.037
A10. You recognize the necessity of perfecting preoperative color Doppler ultrasound examinations for both lower extremities. (P)	92 (90.20)	114(95.00)	0.168

A11. You have placed significant emphasis on monitoring D-dimer levels in the coagulation profile. (P)	96 (94.12)	110(91.67)	0.482

*Positivity is defined as participants selecting options rated 4 or 5.

Table S7. Positive rate of practices of surgeons and nurses in the prevention and cure of PPE after thoracic surgery

	Surgeons (n=102)	Nurses (n=119)	P
P1. How frequently do you actively acquire knowledge pertaining to the prevention and treatment of pulmonary embolism following thoracic surgery through various means, such as engaging in training, reviewing medical literature or expert consensus, and engaging in discussions with fellow medical professionals?	60 (58.82)	50(41.67)	0.011
P2. How often do you evaluate or document risk factors or indications of pulmonary embolism in patients following thoracic surgery prior to the surgical procedure?	69 (67.65)	62(51.67)	0.016
P3. Throughout the course of thoracic surgery, do you conscientiously monitor variables including surgical duration, hematoma compression, and other intraoperative factors, and integrate them into your subsequent practices for the prevention and treatment of pulmonary embolism in patients?	71 (69.61)	69(57.50)	0.062

P4. While performing thoracic surgery, it is essential to maintain a heightened awareness of high-risk factors associated with pulmonary embolism linked to anesthesia, such as limb hypoperfusion and reduced venous blood flow, and incorporate these considerations into your preventative and treatment measures for embolization.	71 (69.61)	68(56.67)	0.047
P5. How closely do you monitor the frequency of symptoms related to pulmonary embolism, such as the pulmonary embolism triad, in post-surgery patients?	79 (77.45)	82(68.33)	0.129
P6. What is the frequency of your clinical engagement in preventative and treatment practices for pulmonary embolism using medications like heparin and warfarin for patients?	76 (74.51)	89(74.17)	0.953
P7. Have you employed techniques such as color Doppler echocardiography and lower limb vascular ultrasonography, as well as other imaging methods, to detect the occurrence of venous thrombosis in patients?	73 (71.57)	74(61.67)	0.120
P8. How frequently do you recommend or assist patients in post-surgery activities such as mobilization, local limb massage, regular repositioning, and elevation of the lower limbs as part of pulmonary embolism prevention and treatment?	87 (85.29)	110(91.67)	0.134

P9. Summarize your experience in the prevention and treatment of pulmonary embolism, encompassing preoperative assessment, intraoperative vigilance, and postoperative care. Apply this experience to enhance the frequency of pulmonary embolism prevention and treatment following thoracic surgery in the future.	79 (77.45)	89(74.17)	0.570

*Positivity is defined as participants selecting options rated 4 or 5.
